# Early [^18^F]FET-PET in Gliomas after Surgical Resection: Comparison with MRI and Histopathology

**DOI:** 10.1371/journal.pone.0141153

**Published:** 2015-10-26

**Authors:** Benjamin Kläsner, Niels Buchmann, Jens Gempt, Florian Ringel, Constantin Lapa, Bernd Joachim Krause

**Affiliations:** 1 Department of Nuclear Medicine, Klinikum rechts der Isar der TU München, Ismaninger Str. 22, 81675, München, Germany; 2 Department of Neurosurgery, Klinikum rechts der Isar der TU München, Ismaninger Str. 22, 81675, München, Germany; 3 Department of Nuclear Medicine, Klinikum Konstanz, Luisenstr. 7a, 78464, Konstanz, Germany; 4 Department of Nuclear Medicine, Universitätsklinikum Würzburg, Oberdürrbacher Str. 6, 97080, Würzburg, Germany; 5 Department of Nuclear Medicine, Universitätsmedizin Rostock, Gertrudenplatz 1, 18057, Rostock, Germany; Glasgow University, UNITED KINGDOM

## Abstract

**Background:**

The precise definition of the post-operative resection status in high-grade gliomas (HGG) is crucial for further management. We aimed to assess the feasibility of assessment of the resection status with early post-operative positron emission tomography (PET) using [^18^F]O-(2-[^18^F]-fluoroethyl)-L-tyrosine ([^18^F]FET).

**Methods:**

25 patients with the suspicion of primary HGG were enrolled. All patients underwent pre-operative [^18^F]FET-PET and magnetic resonance imaging (MRI). Intra-operatively, resection status was assessed using 5-aminolevulinic acid (5-ALA). Imaging was repeated within 72h after neurosurgery. Post-operative [^18^F]FET-PET was compared with MRI, intra-operative assessment and clinical follow-up.

**Results:**

[^18^F]FET-PET, MRI and intra-operative assessment consistently revealed complete resection in 12/25 (48%) patients and incomplete resection in 6/25 cases (24%). In 7 patients, PET revealed discordant findings. One patient was re-resected. 3/7 experienced tumor recurrence, 3/7 died shortly after brain surgery.

**Conclusion:**

Early assessment of the resection status in HGG with [^18^F]FET-PET seems to be feasible.

## Introduction

The importance of complete resection of contrast-enhancing tumor in patients with malignant gliomas has been highlighted by several studies [1, 2]. The precise definition of the early post-operative resection status in high-grade gliomas (HGG) is crucial for further treatment planning, e.g. delineation of the radiation field in radiotherapy or surgical re-evaluation if larger residual tumor is present.

Magnetic resonance imaging (MRI) represents the state-of-the-art technique for brain tumor imaging. T1- and T2-weighted sequences provide information on glioma size and localization as well as additional insights on secondary phenomena, i.e. edema, bleeding, necrosis and signs of increased intracranial pressure [3, 4]. In the early postoperative setting after brain tumor surgery, MRI is the imaging modality of choice to verify the resection status. However, the time window for imaging is restricted, because reactive tissue changes normally start to evolve after 48–72 hours.

Many studies have demonstrated the value of [^18^F]O-(2-[^18^F]-fluoroethyl)-L-tyrosine positron emission tomography ([^18^F]FET-PET) for delineation of tumor tissue [5–8] and definition of the optimal biopsy site whereas MRI changes may be unspecific and exceed true tumor boundaries (3–5). Additionally, malignant high-grade gliomas can present without contrast enhancement and may thereby be underestimated by MRI [5, 8–10]. In contrast, amino acid tracer uptake even in non-solid, infiltrative parts of gliomas has been demonstrated [11, 12]. Moreover, [^18^F]FET-PET offers a superior differentiation between tumor tissue and reactive changes as it does not accumulate in inflammatory or reactive processes [13, 14]. It improves tumor recurrence detection and results in a better definition of target volumes prior to radiation therapy [15–17].

In this study we investigated the value of early post-operative [^18^F]FET-PET to assess the resection status in comparison to intra-operative findings as well as MRI.

## Patients and Methods

### Patients

Twenty five consecutive patients (16 males, 9 females; mean age, 62±14 y [range, 26–78 years]) with suspected primary HGG were prospectively enrolled in this study. Tumor surgery was performed in all subjects using dedicated neuro-navigation software (iPlan 3.0; BrainLab, Feldkirchen; Germany), in eloquent localizations with intraoperative neuromonitoring by somatosensory evoked potentials (SSEP) and motor evoked potentials (MEP). Tumor tissue was removed using the cavitron ultrasonic surgical aspirator (CUSA, Valleylab Boulder, CO, USA) device and specimens for histopathologic diagnosis were obtained from the central parts of the tumor. No carmustine wafers were introduced into the resection cavity.

All patients underwent pre- and post-operative [^18^F]FET-PET and MRI. Intra-operative assessment of tumor resection with 5-ALA-induced fluorescence and clinical follow-up (including imaging) served as additional reference standards. The study was approved by the local ethics committee of Technical University, Munich, Germany. Written informed consent was obtained from all patients.

### Imaging protocols

The PET studies were performed prior to and after (mean, 3±2 days; median, 2 days) the resection using a dedicated ECAT EXACT HR+ PET scanner (Siemens CTI, Knoxville, TN, USA) in 3-D mode. 182±34 MBq of [^18^F]FET were intravenously injected. Patients were positioned supine using a low-attenuation head holder. Static acquisitions from 20–40 min p.i. followed by a 7 minute transmission scan using [^68^Ga]/[^68^Ge] rod sources were performed. Images were reconstructed using 3D filtered backprojection with a Hann filter (cut off frequency 0.34) and corrected for attenuation, scatter and randoms.

MRI was performed on a Philips Achieva 3.0 MR-tomograph (Philips Healthcare, Amsterdam, The Netherlands). Patients were positioned supine. Pre-operatively, axial T1±Gd, T2 FLAIR, T2*, DWI with ADC map and 3D T1+Gd for navigation sequences were acquired.

After resection (mean; 1±1 day; median; 1 day), axial T1±Gd and subtraction, T2 FLAIR and T2* sequences were obtained. Follow-up MRI and/or PET scans were performed at intervals of three months or at clinical suspicion of tumor recurrence/progression.

### Data analysis

PET:

Pre-operative scans served as reference for post-operative imaging. Analysis was performed by visual comparison (B.K., C.L.) of both data sets. Focal [^18^F]FET uptake in the primary tumor region higher than background was considered positive for tumor tissue.

MRI:

All images were analyzed independently by an experienced neuroradiologist who was blinded to the PET data. Residual tumor was defined as previously described [18].

### Histopathology and Immunohistochemistry

All tumor samples were histologically assessed and graded using standard hematoxylin and eosin (H&E) sections (3–4 μm) according to the 2007 World Health Organisation criteria [19]. The astrocytic origin of the tumors was confirmed by positive immunoreaction for the glial fibrillary acidic protein (GFAP 1:200, Dako, Glostrup, Denmark). Oligodendroglial features were assured by the distinct pattern of microtubule-associated protein 2 immunoreactivity (MAP2 1:250, Dako, Glostrup, Denmark).

## Results

### Tumor characteristics

All patients showed contrast-enhancing brain tumors in pre-operative MRI. Histopathology confirmed HGG in 21/25 patients: 18/25 had glioblastoma, 3 patients anaplastic oligoastrocytoma. 2 patients suffered from grade II oligodendroglioma, the remaining 2 from grade II astrocytoma. Information on the patients´ characteristics is provided in Table 1.

**Table 1:** Patients´ characteristics.

Patient	Age (in years)	Gender	Histopathology
**1**	58	Male	Glioblastoma
**2**	37	Female	Anaplastic oligoastrocytoma III
**3**	60	Male	Glioblastoma
**4**	76	Female	Oligodendroglioma II
**5**	64	Male	Glioblastoma
**6**	69	Male	Glioblastoma
**7**	63	Male	Glioblastoma
**8**	41	Male	Astrocytoma II
**9**	70	Female	Glioblastoma
**10**	68	Male	Glioblastoma
**11**	64	Male	Glioblastoma
**12**	23	Male	Astrocytoma II
**13**	52	Male	Anaplastic oligoastrocytoma III
**14**	56	Male	Glioblastoma
**15**	75	Male	Glioblastoma
**16**	73	Male	Glioblastoma
**17**	56	Male	Glioblastoma
**18**	65	Male	Glioblastoma
**19**	52	Female	Oligodendroglioma II
**20**	63	Female	Glioblastoma
**21**	79	Female	Glioblastoma
**22**	46	Female	Glioblastoma
**23**	69	Female	Glioblastoma
**24**	37	Female	Anaplastic oligoastrocytoma III
**25**	57	Male	Glioblastoma

### [^18^F]FET-PET imaging results in comparison to the reference standards

**Fig 1:**
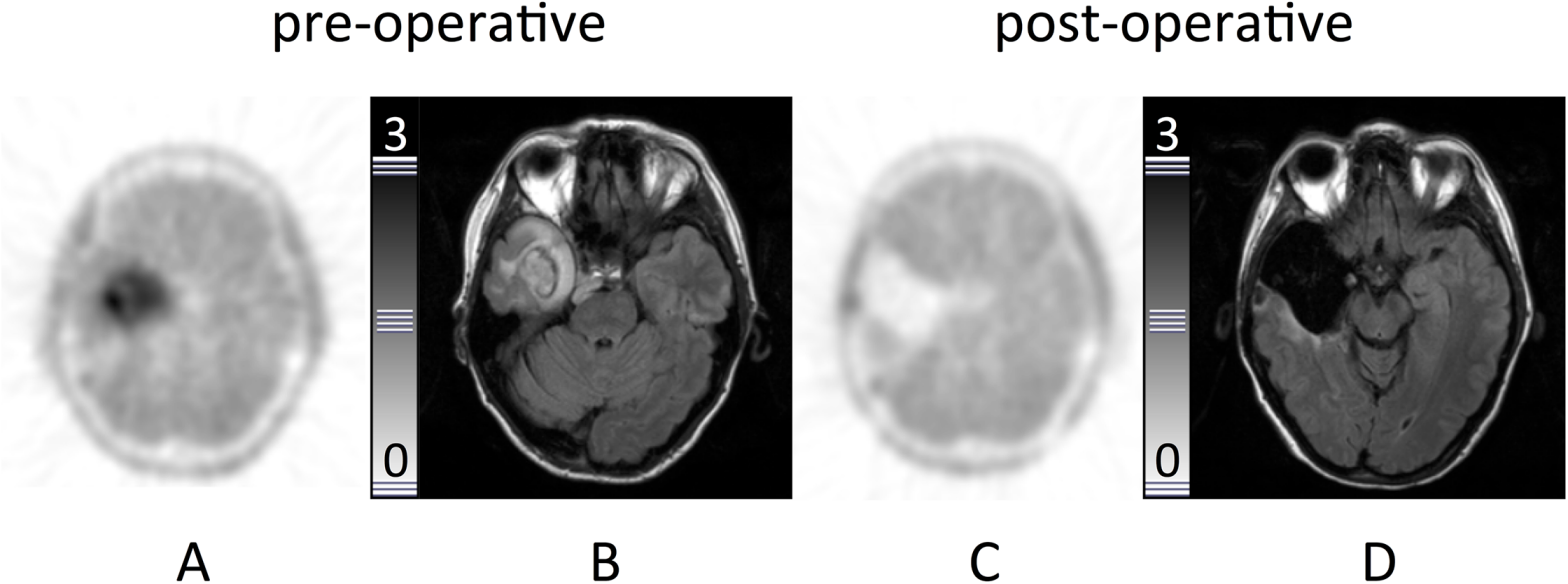
Example of a complete resection. Patient #2. Transaxial slices. **A:** Intense focal [^18^F]FET uptake in the right temporal lobe. **B:** MRI (FLAIR-sequence) with diffuse hyperintensity in the same region. **C:** [^18^F]FET-PET (48h after resection) and **D:** MRI (24 h after resection, FLAIR) with no signs of residual tumor tissue.

Intra-surgical assessment of resection status using 5-ALA postulated complete resection in 17/25 [76%) of cases. Post-operative MRI was concordantly negative for remaining tumor in 17/25 subjects. In one patient, it was negative despite intra-operative concern for incomplete resection, in another case it detected contrast enhancement highly suspicious for vital tumor in contrast to a negative surgical report. In 12/25 patients (48%) post-operative [^18^F]FET-PET revealed no remaining tumor tissue consistent with the two other reference modalities. An example is given in Fig 1. In the remaining 13 subjects (52%), focally increased [^18^F]FET uptake suspicious for tumor remnants was observed. 6 out of those 13 cases (46%) were consistent with both MRI and 5-ALA and therefore considered true positive.

**Fig 2:**
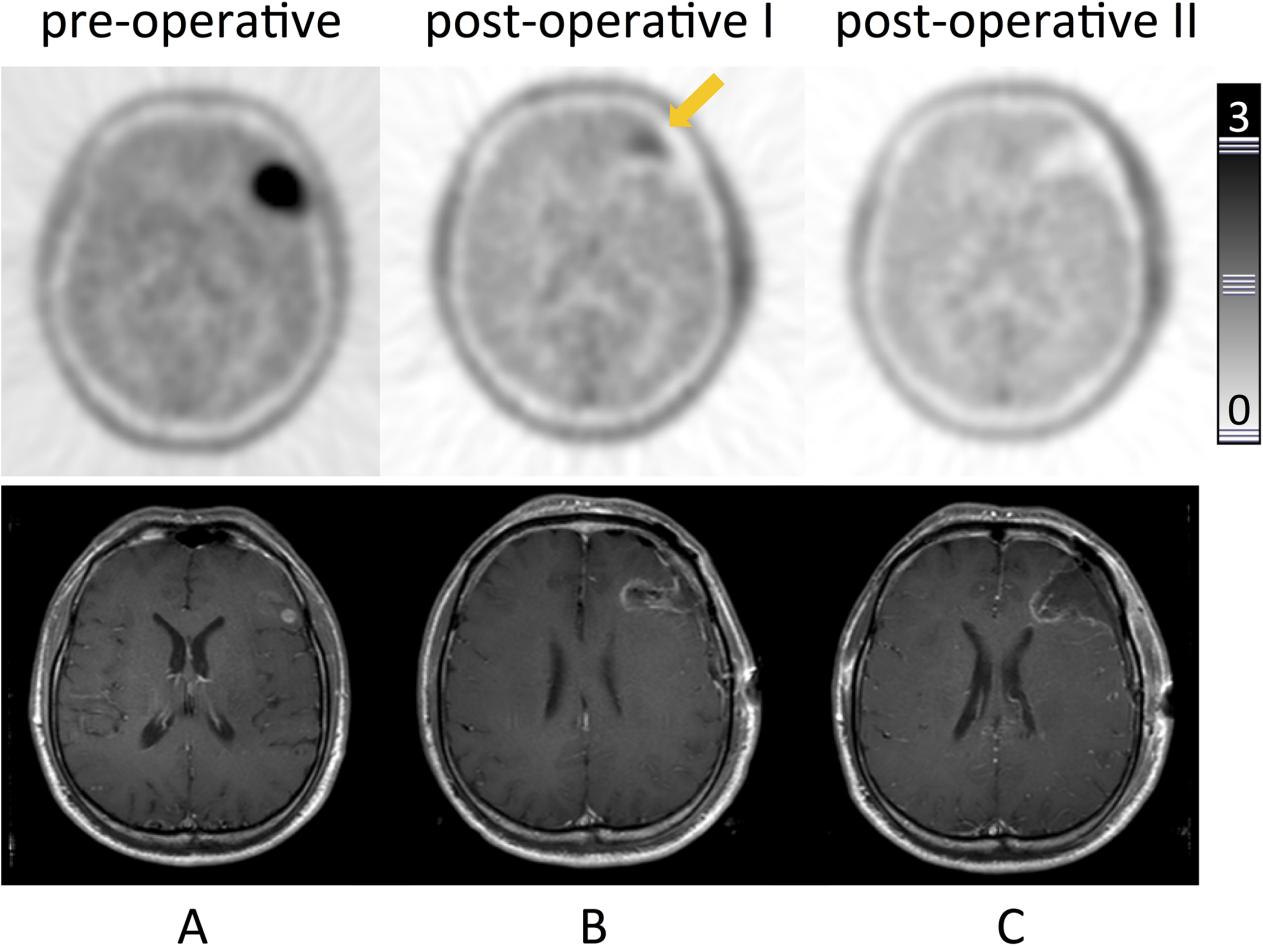
Residual glioma tissue detected by [^18^F]FET-PET–Re-resection. Patient #7. Transaxial slices of [^18^F]FET-PET (upper row) and corresponding contrast-enhanced T1-weighted MRI. **A:** Pre-operative PET with intense focal uptake in the left frontal lobe consistent with a lesion on MRI. **B:** Early postoperative PET with focal uptake at the cranio-medial border of the resection cavity, leading to re-resection; MRI displaying unspecific changes. **C:** Early postoperative PET after re-resection showing the resection cavity with no focal uptake in the border region, consistent with complete resection (confirmed by MRI). Histopathology confirmed glioblastoma.

**Fig 3:**
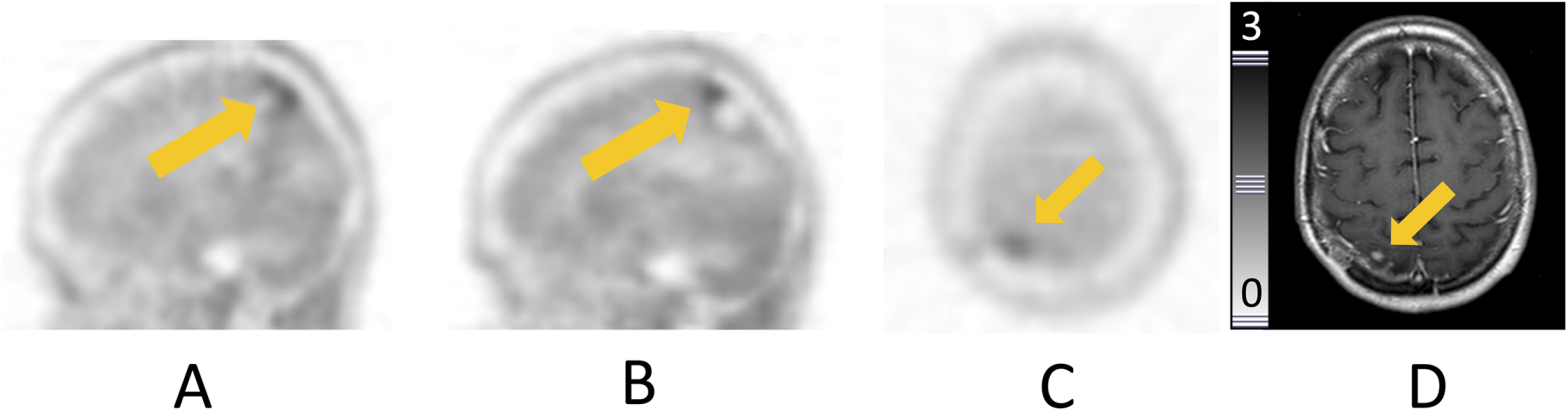
Residual tumor tissue detected by [^18^F]FET-PET—Follow-up. Patient #18. **A,B:** sagittal slices. **C,D:** axial slices. **A:** Pre-operative PET showing focal uptake in the right parietal lobe. **B:** Suspicious uptake in the cranial border of the resection cavity 48h and **C:** 4 months after resection, consistent with vital tumor. **D:** Corresponding MRI 4 months after resection corroborating the PET finding.

In 7 patients, PET revealed discordant findings. In one subject (#15), both PET and 5-ALA were positive for residual tumor whereas MRI did not indicate tumor tissue. This patient died only three months after brain surgery. In another patient (#23), both imaging modalities raised the concern for viable tumor despite negative intra-surgical assessment. This patient deceased five months later. In the remaining 5 patients (#7, 16, 18, 21, 22), PET was the only modality to demonstrate tumor remnants. One patient (#7) was suitable for re-resection which was performed 48 h after the first surgery. Histopathology confirmed vital tumor tissue exactly at the spot of maximum [^18^F]FET-PET uptake (Fig 2). All other lesions detected by [^18^F]FET-PET could not be re-resected due to tumor localization and were followed-up. After three months, recurrence exactly at the site of initial tracer accumulation in post-operative PET was detected in three of the five patients (#16, 18, 22; Fig 3). All patients underwent radiation therapy. The remaining patient (#21) died three months after surgery. Table 2 gives an overview over the imaging results of the individual patients.

**Table 2:** Comparison of all 3 modalities: PET, MRI and intraoperative findings (IF).

Patient	Resection status			Miscellaneous
	**IF**	**MRI**	**PET**	
**1**	IR	IR	IR	-
**2**	CR	CR	CR	-
**3**	CR	CR	CR	-
**4**	CR	CR	CR	-
**5**	CR	CR	CR	-
**6**	CR	CR	CR	-
**7**	CR	CR	IR	Re-resection
**8**	CR	CR	CR	-
**9**	IR	IR	IR	-
**10**	CR	CR	CR	-
**11**	IR	IR	IR	-
**12**	CR	CR	CR	-
**13**	CR	CR	CR	-
**14**	IR	IR	IR	-
**15**	IR	CR	IR	Death after 3 months
**16**	CR	CR	IR	Recurrence at PET site
**17**	IR	IR	IR	-
**18**	CR	CR	IR	Recurrence at PET site
**19**	CR	CR	CR	-
**20**	CR	CR	CR	-
**21**	CR	CR	IR	Death after 3 months
**22**	CR	CR	IR	Recurrence at PET site
**23**	CR	IR	IR	Death after 5 months
**24**	CR	CR	CR	-
**25**	IR	IR	IR	-

**IR** = incomplete resection; **CR** = complete resection.

## Discussion

The aim of this pilot study was to assess the value of early post-operative [^18^F]FET-PET in the detection of residual tumor tissue in HGG patients. PET results were compared to MRI, intra-operative reports and histopathology.

While various studies have demonstrated the value of amino-acid based PET with both [^11^C]-methionine and [^18^F]FET in brain tumors including the detection of primary [14, 20–22] and recurrent disease [15, 23], the differentiation between radiation necrosis versus tumor recurrence [24], as well as therapy monitoring (16), this is the first study to investigate the performance of early post-operative [^18^F]FET-PET.

Our data suggest that PET can detect vital residual tumor tissue with high accuracy. All patients with complete resection were correctly classified by PET, thus proving a high negative predictive value. Interestingly, the rate of complete resections of this series was quite high with 75%, but might be explained by the multi-modality approach prior to surgery as well as beneficial localization of the individual tumors in many cases allowing for unlimited surgical approaches.

Compared to MRI, early post-operative [^18^F]FET-PET showed a comparable accuracy in the evaluation of the resection status. In 19/25, both imaging modalities were in concordance indicating complete (n = 12) and incomplete (n = 7) resection. One patient with glioblastoma in whom both [^18^F]FET-PET and MRI detected vital tumor tissue in discordance to negative intra-operative assessment died only five months after imaging.

Additionally, [^18^F]FET-PET was more sensitive than MRI in 24% (6/25) of cases in which MRI was false negative as proven by histopathology or short-term follow-up. It impacted on patient management in one subject who successfully underwent surgical re-evaluation. These results are in line with a previously published study that referred the possibility of early post-operative PET-imaging with [^11^C]-methionine in glioma patients. Tanaka *et al*. could demonstrate the feasibility of a multimodal navigation system using metabolic ([^11^C]-methionine-PET) and anatomical (MRI) information in glioma patients and reported on tumor remnants exclusively detected by post-operative PET in 2 of 12 cases [25]. Our data add evidence on the suitability of early metabolic imaging after glioma surgery.

There are limitations of this study. First, image guided tissue sampling would have been necessary to validate whether remnants (either in MRI or PET) were truly tumor tissue, especially for conflicting results in the two post-operative imaging modalities. Second, only a small number of patients were included in this pilot study. Third, the problem of brain shift occurring after surgery cannot be completely excluded thereby complicating analysis of early post-operative imaging. Last, histological work-up did not take into account spatial information to correlate imaging findings with surgery.

To date, MRI is the gold standard of brain imaging. In a large study more than 200 patients with newly diagnosed glioblastoma, subjects with residual contrast-enhancing tumor as revealed by early MRI scans had a shorter median overall survival from the time of surgery as compared to patients without residual tumor (1, 2). Detection of residual vital tumor is therefore critical. However, MRI is prone to unspecific changes which can occur early after surgery. Additional PET imaging could raise confidence of diagnosis and impact on adjuvant therapy, e.g. tailored radiotherapy, by guiding a potential boost to tumor areas. Furthermore, early knowledge of the post-operative PET status can prove extremely useful in the setting of follow-up imaging in which the differentiation of true tumor recurrence from pseudo-progression has important therapeutic implications.

In conclusion, early assessment of the resection status in HGG seems to be feasible with [^18^F]FET-PET and might prove helpful for the prediction of residual tumor tissue or early recurrence. Future studies are warranted to further evaluate the value of early post-surgical [^18^F]FET-PET and its implications on patient management.
